# Novel CBG Derivatives Can Reduce Inflammation, Pain and Obesity

**DOI:** 10.3390/molecules26185601

**Published:** 2021-09-15

**Authors:** Natalya M. Kogan, Yarden Lavi, Louise M. Topping, Richard. O. Williams, Fiona E. McCann, Zhanna Yekhtin, Marc Feldmann, Ruth Gallily, Raphael Mechoulam

**Affiliations:** 1 Medical Faculty, Institute for Drug Research, Hebrew University, Jerusalem 91120, Israel; yarden.lavi@mail.huji.ac.il (Y.L.); raphaelm@ekmd.huji.ac.il (R.M.); 2 Kennedy Institute of Rheumatology, University of Oxford, Oxford OX3 7FY, UK; louise.topping@kennedy.ox.ac.uk (L.M.T.); richard.williams@kennedy.ox.ac.uk (R.O.W.); marc.feldmann@kennedy.ox.ac.uk (M.F.); 3 180 Life Sciences, Menlo Park, CA 94025, USA; fmccann@180lifesciences.com; 4 Lautenberg Center of Immunology and Cancer Research, The Hebrew University of Jerusalem, Jerusalem 91120, Israel; zhannay@savion.huji.ac.il (Z.Y.); ruthg@ekmd.huji.ac.il (R.G.)

**Keywords:** cannabinoid, cannabigerol, anti-inflammatory, obesity

## Abstract

Interest in CBG (cannabigerol) has been growing in the past few years, due to its anti-inflammatory properties and other therapeutic benefits. Here we report the synthesis of three new CBG derivatives (HUM-223, HUM-233 and HUM-234) and show them to possess anti-inflammatory and analgesic properties. In addition, unlike CBG, HUM-234 also prevents obesity in mice fed a high-fat diet (HFD). The metabolic state of the treated mice on HFD is significantly better than that of vehicle-treated mice, and their liver slices show significantly less steatosis than untreated HFD or CBG-treated ones from HFD mice. We believe that HUM-223, HUM-233 and HUM-234 have the potential for development as novel drug candidates for the treatment of inflammatory conditions, and in the case of HUM-234, potentially for obesity where there is a huge unmet need.

## 1. Introduction

CBG (cannabigerol) was discovered by Gaoni and Mechoulam in cannabis resin (hashish) in 1964 and was considered a missing link in the biosynthesis of THC (tetrahydrocannabinol) [1]. Cannabinoid biosynthesis begins with the combination of geranyl pyrophosphate and olivetolic acid to form CBGA (Cannabigerolic acid). CBGA serves as the substrate for the synthesis of Δ9-THCA (THC acid) and CBDA (cannabidiolic acid). Decarboxylation of CBGA, Δ9-THCA, and CBDA by heat results in CBG, Δ9-THC, and CBD (cannabidiol), respectively. Because CBGA serves as the substrate for the synthesis of the major cannabinoids, very little is typically found naturally in material from Cannabis sp. [2].

While the cannabis constituents CBD and THC have been thoroughly investigated [3,4], research on CBG has been relatively neglected. Based on established pharmacological properties, there is growing evidence that CBG has therapeutic potential for treating neurological disease, gastrointestinal disease as well as some metabolic disorders [2,5,6,7,8,9,10,11,12]. Notably, having mechanisms both in common and distinct from THC and CBD respectively, it was of interest to explore the anti-inflammatory and analgesic properties of CBG, that may be relevant in the aforementioned disease areas.

Indeed, CBG has been shown to exert anti-inflammatory effects and some derivatives of CBG have been synthesized and tested in both animal models and human patient [5,6,7,8,9]. CBG has also been proved to possess anti-inflammatory properties in neurological models [6,7,13] and confer other therapeutic benefits such as appetite stimulation [14]. In addition, CBG is known to activate α2-adrenoreceptor [15] and to interact with sub-types of TRPV (Transient Receptor Potential Vanilloid) channels, pertinent to signaling associated with gastrointestinal inflammation [16]. Association with the classical cannabinoid receptors CB1 and CB2R has been determined, with binding demonstrated to modulate signaling mediated by receptors and receptor heteromers even at low concentrations of 0.1–1 μM [17]. There have been some attempts to investigate and expand CBG’s SAR (Structure-Activity Relationship) and these investigations have led to novel pharmaceutical candidates for Parkinson’s disease [6,8].

The immune and inflammatory system has evolved to protect against foreign organisms and substances, antigens, that may cause damage to the normal function of the body. The immune response, when dysregulated, may also lead to various pathological conditions, including tumors [18], autoimmune diseases [19], obesity [20], diabetes [21], cardiovascular diseases [22] and more. Western medicine has introduced an array of drugs aiming at the immune system trying to manipulate its response and reducing side effects of acute inflammation. Among the most common medications used by the public are corticosteroids and Non-steroidal Anti-inflammatory Drugs (NSAIDs). Despite being the universal first choice in treating inflammatory diseases, these drug classes have long been known for their adverse side effects, limiting their dosage and prolonged treatment regimens [23,24,25,26,27].

Here we report the synthesis of three new synthetic CBG derivatives and show them to possess anti-inflammatory and pain-resolving properties in preclinical models. In addition, one of these molecules, HUM-234, has also shown prominent activity in obesity prevention in mice fed a high-fat diet (HFD). Importantly, the metabolic state of the HUM-234 treated HFD mice is significantly closer to healthy levels than that of vehicle-treated HFD mice, and their liver slices show much less steatosis than untreated, or CBG-treated livers from HFD mic, suggesting that HUM-234 and related compounds may have potential to treat metabolic disorders including liver disease.

## 2. Results

### 2.1. Chemistry Development and Synthesis of HUM-223, HUM-233 and HUM-234

**Figure 1:**
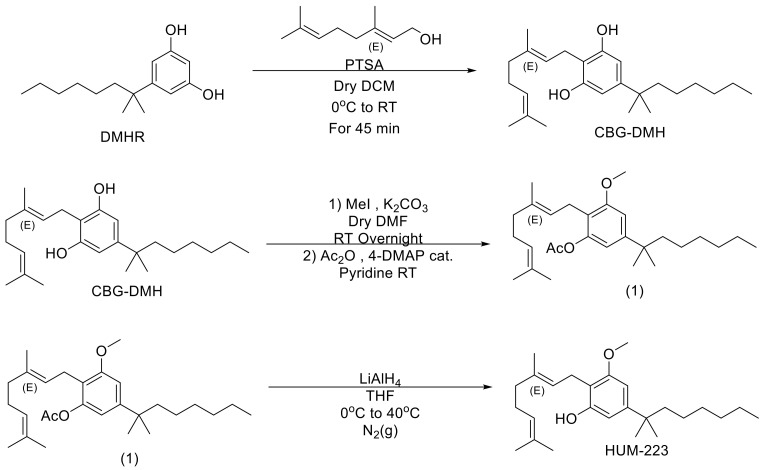
The preparation of HUM-223.

We sought to explore the effect of a bulky residue at position 5 of CBG in place of the natural pentyl on its anti-inflammatory activity. This is based on similar observations made for THC, among other cannabinoids, in which a bulkier residue such as dimethylheptyl (DMH) resulted in an improved efficacy at the relevant biological model [28]. We have also seen in our previous work with CBD derivatives that bulkier DMH derivatives were more active in the knee arthritis model (unpublished results). We have therefore prepared compound HUM-223 which is a monomethoxy CBG-DMH (Figure 1). During the preparation, the partial reaction with iodomethane resulted in a mixture of fully reacted dimethoxy product, partially reacted monomethoxy and an unchanged CBG-DMH. This mixture was hard to isolate on its own and we, therefore, decided to add an acetylation step to the preparation. The crude mixture of the iodomethane reaction was reacted with an excess of acetic anhydride. This made the chromatography easier and allowed us to isolate the pure monomethoxylated product (1). The acetate protecting group can then easily be removed using LiAlH_4_ (Lithium Aluminium Hydride) to produce the final HUM-223.

**Figure 2:**
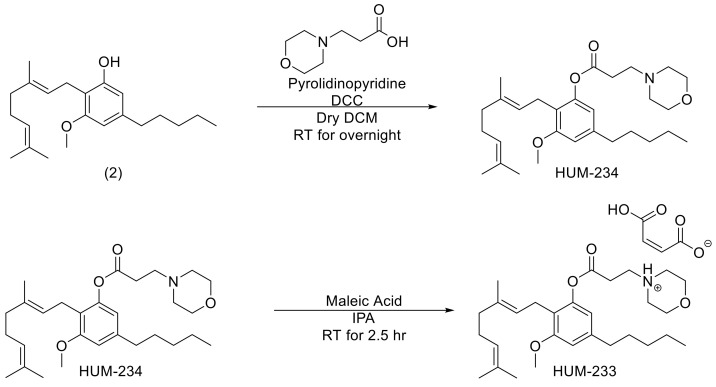
The synthesis of HUM-234 and its maleate salt HUM-233 from monomethoxy CBG.

Another alteration deemed to be useful, namely the use of a morpholine propionate ester at one of the phenol positions of CBG. This concept is based on observations made by our group for a similar modification of CBD, which produced an improvement in the anti-inflammatory properties [29]. Coupling of monomethoxy CBG (2) with morpholinopropionic acid yielded therefore compound HUM-234 (Figure 2).

To further improve its bioavailability, HUM-234 was also turned into a maleic acid salt named HUM-233 (Figure 2). This was based on a similar modification previously carried out by our group with CBD [29]. Since maleic acid has no inherent anti-inflammatory activity, it serves only to increase the compound’s bioavailability. As HUM-233 is solid at room temperature, whereas HUM-234 is an oil, we believe that this could offer an additional benefit should this compound prove efficacious since it is easier to handle solid compounds when preparing pharmaceuticals, making the measurement of dose more accurate.

### 2.2. Evaluation of Anti-Inflammatory Activity

**Figure 3:**
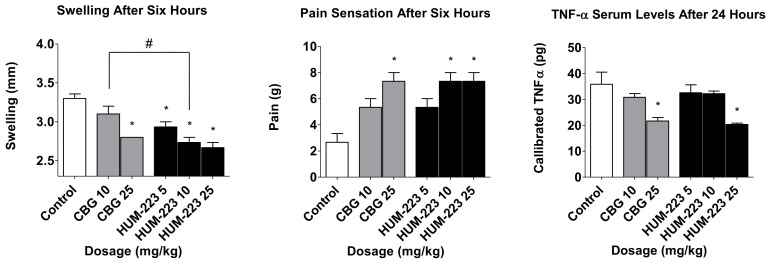
Anti-inflammatory and analgesic evaluation of HUM-223 (black) compared to CBG (cannabigerol) (grey) and vehicle control (white). Statistical comparison was done by 1-way ANOVA (*p*-value = 0.0002) and post hoc analysis by Tukey’s test. * *p* < 0.05 comparing to control group. # *p*-value < 0.05 in the indicated comparison.

HUM-223 was compared to CBG and to a vehicle control group in three inflammation assays: paw swelling, pain sensation in the paw and circulating TNF-α (Figure 3). CBG itself did not show a consistent pattern of efficacy in all three assays, unfortunately. However, when comparing HUM-223 to CBG we observed significant improvement in the paw swelling assay at a dose of 10 mg/kg.

**Figure 4:**
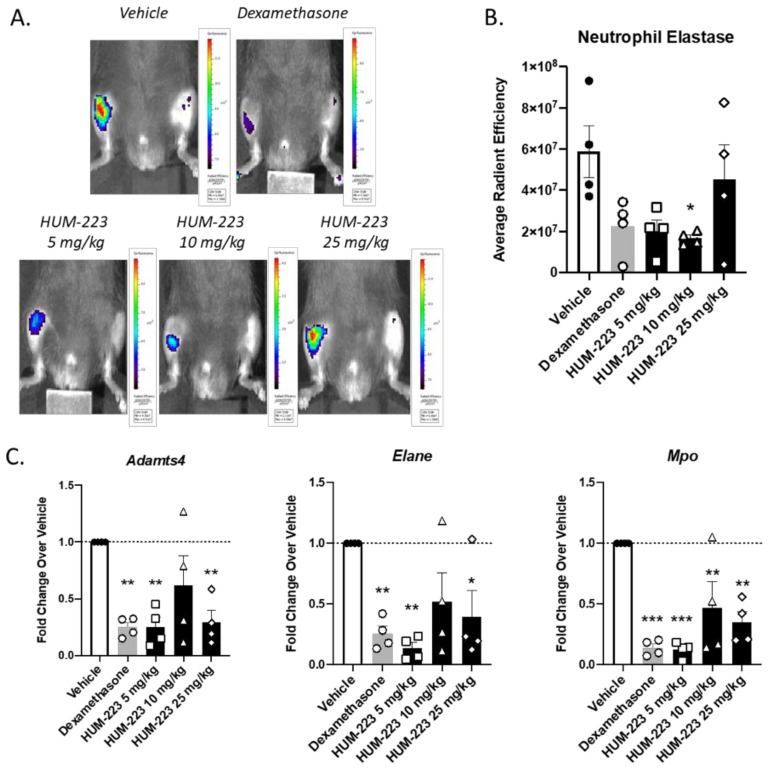
Anti-inflammatory effect of HUM-223 in zymosan-induced arthritis. Mice were treated with vehicle (white bars), dexamethasone (2 m/kg, grey bars) or HUM-223 (black bars). (**A**) Representative images of neutrophil elastase IVIS fluorescence imaging of knees of ZIA mice treated with vehicle control or HUM-223. (**B**) Quantification of neutrophil elastase average radiant efficiency in the inflamed knee of ZIA mice. (**C**) Gene expression analysis of inflamed ZIA knee joints. Statistical analysis by one-way ANOVA (*p*-value 0.0392 for Neutrophil elastase IVIS, 0.0080 for Elane, 0.0037 for Adams4 and 0.0002 for MPO) with Tukey’s post-hoc test. * *p* < 0.05, ** *p* < 0.001, *** *p* < 0.0001 compared to vehicle-treated mice.

The anti-inflammatory ability of HUM-223 was also assessed in zymosan-induced arthritis (ZIA), a model of acute inflammation in the knee (Figure 4), in comparison to dexamethasone. This molecule has been chosen for knee arthritis assay as previously CBD-DMH derivatives have shown better results in this model than pentyl-chain derivatives. HUM-223 significantly reduced neutrophil elastase levels at 10 mg/kg, in a manner comparable to dexamethasone, when compared to vehicle only treated mice, as measured by in vivo imaging of a fluorescent reporter (IVIS). Additionally, gene analysis of inflamed knee joints demonstrated the ability of HUM-223 to significantly reduce expression of pro-inflammatory genes which encode for enzymes that degrade matrix proteins important for structural integrity, namely adamts4 (A disintegrin and metalloproteinase with thrombospondin motifs 4), neutrophil elastase (Elane) and myeloperoxidase (Mpo), with 5 mg/kg having the greatest effect.

**Figure 5:**
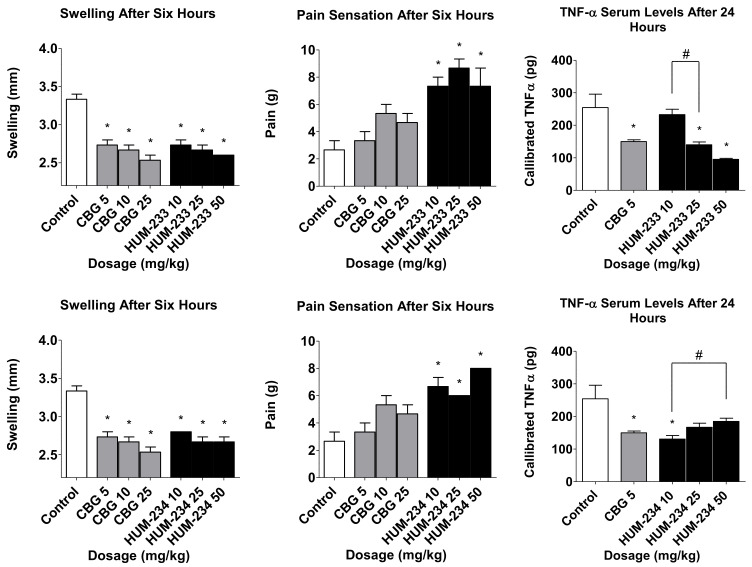
Anti-inflammatory and analgesic evaluation of HUM-233 and HUM-234 (black) compared to CBG (grey) and vehicle control (white). Statistical comparison was carried out by one-way ANOVA (*p*-value < 0.0001) and post hoc analysis by Tukey’s test. * *p* < 0.05 comparing to control group. # *p* < 0.05 in the indicated comparison.

The biological results revealed that both HUM-233 and HUM-234 have anti-inflammatory activity (Figure 5). Both compounds showed improvement of swelling and pain sensation which is comparable to CBG in all tested doses. HUM-233 was able to reduce TNF-α levels in doses of 25 and 50 mg/kg and presented a distinct increase of biological reaction when the doses were increased. HUM-234 showed an opposite trend at elevated doses. HUM-233 and HUM-234 were comparable but were not significantly better than CBG in these assays.

### 2.3. Evaluation of Effects on Weight Gain

**Figure 6:**
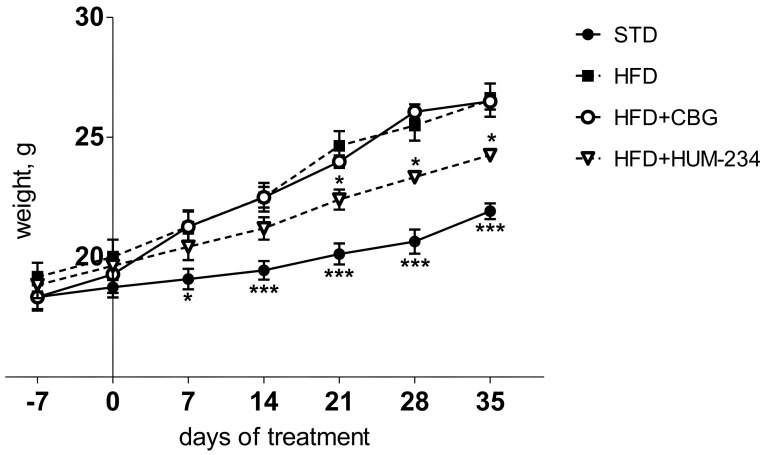
Anti-obesity evaluation of HUM-234 (25 mg/kg) in comparison to CBG (15 mg/kg), HFD (high fat diet) and STD (standard food diet). Statistical analysis by two-way ANOVA matching treatment groups in different days (*p*-value < 0.0001). Post-hoc analysis by Bonferroni test. * *p* < 0.05, *** *p* < 0.001 comparing to HFD group.

**Figure 7:**
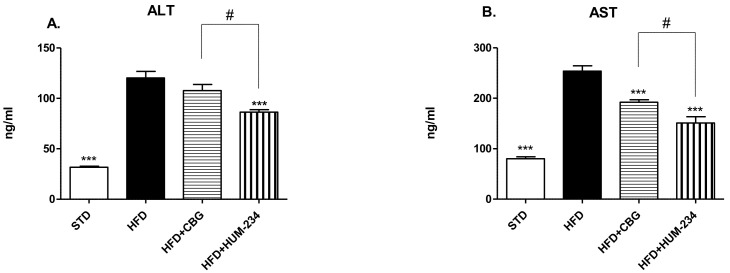
Liver enzymes evaluation of HUM-234 (25 mg/kg) in comparison to CBG (15 mg/kg), HFD (high fat diet) and STD (standard food diet). (**A**) ALT (Alanine transaminase) (**B**) AST (Aspartate transaminase). Statistical analysis by one-way ANOVA (*p*-value < 0.0001 for both ALT and AST) and post hoc analysis by Tukey’s test. *** *p* < 0.001 comparing to HFD group. # *p*-value < 0.05 in the indicated comparison.

**Figure 8:**
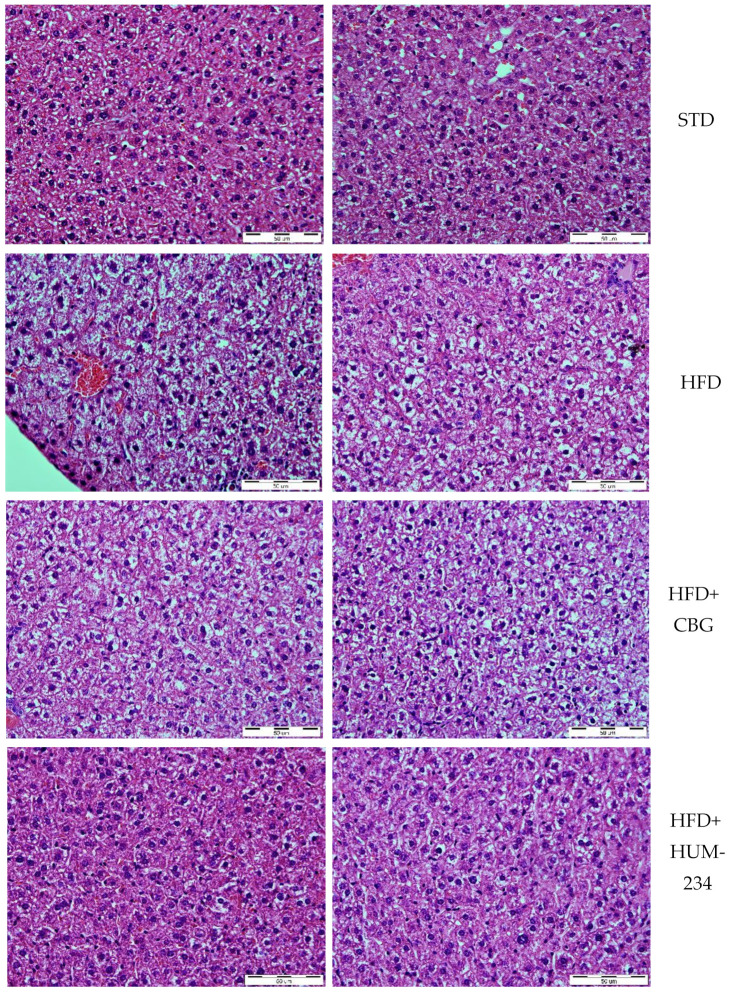
Liver steatosis in HUM-234 (25 mg/kg) in comparison to CBG (15 mg/kg), HFD (high-fat diet) and STD (standard food diet). Magnification 40×, bar length 50 µm.

Despite having comparable anti-inflammatory activity, HUM-234 is much more active than CBG in the prevention of obesity. Adult female mice fed an HFD (high-fat diet) gradually gain weight, much faster than STD (standard diet) fed ones. CBG (15 mg/kg, the dose which caused the least weight gain from the preliminary experiments) does not limit weight gain in our model. On the contrary, the weight gain is at some time points even higher for this group than for the vehicle-treated HFD group. However, HUM-234-treated mice (25 mg/kg) gain weight much slower than HFD or HFD + CBG groups (Figure 6). The ALS (Alanine Transaminase) and AST (Aspartate Transaminase) enzymes levels are elevated in the HFD group comparing to STD; HUM-234 significantly reduces their levels (Figure 7), significantly better than CBG. Liver slices of the HFD mice show liver steatosis (liver cells are not dense with large white fat areas between them), while slices of HUM-234-treated mice livers show almost no steatosis (liver cells are dense with almost no white fat areas between them), comparable to healthy livers from mice on the standard diet (STD group, Figure 8); the livers of CBG-treated mice show almost as much steatosis as those of HFD mice.

It is of great interest that while the anti-inflammatory and pain-relieving activity of CBG and HUM-234 are similar, HUM-234 ameliorates weight gain, in contrast to CBG, suggesting this compound has the potential for development as an anti-obesity drug.

## 3. Discussion

Here we report the synthesis of, and the evaluation in-vivo of the anti-inflammatory, analgesic and anti-obesity of three novel CBG derivatives.

Inflammation contributes to the pathogenesis of many diseases. Chronic inflammation can lead to cardiovascular diseases, gastrointestinal diseases, obesity, asthma, arthritis, neurodegenerative diseases, cancer and more.

Currently, the drugs used to treat inflammation are most often NSAIDs and steroids. Monoclonal antibodies to cytokines, TNF, IL6 and IL12/23 are used if these do not work. NSAIDs work by inhibiting the activity of cyclooxygenase enzymes (COX-1 or COX-2). In cells, these enzymes are involved in the synthesis of prostaglandins, which are associated with inflammation, and thromboxanes, which are involved in blood clotting [30]. Most NSAIDs are non-selective and inhibit the activity of both COX-1 and COX-2. These NSAIDs, while reducing inflammation, also inhibit platelet aggregation and increase the risk of gastrointestinal ulcers/bleeds [31]. Side effects can include an increased risk of gastrointestinal ulcers and bleeds, heart attack, and kidney disease [32,33]. COX-2 selective inhibitors have fewer gastrointestinal side effects but promote thrombosis and some of these agents substantially increase the risk of heart attack [31]. By inhibiting physiological COX activity, all NSAIDs increase the risk of kidney disease [34] and through a related mechanism, heart attack. In addition, NSAIDs can blunt the production of erythropoietin resulting in anemia, since hemoglobin synthesis depends on this hormone [35].

Of equal if not greater concern are side effects associated with long-term use of steroidal drugs. Although highly effective in the elimination of inflammation, they can cause obesity, growth retardation in children, and even lead to convulsions and psychiatric disturbances, osteoporosis, adrenal suppression, hyperglycemia, dyslipidemia, cardiovascular disease, Cushing’s syndrome, immunosuppression. an increase in the rate of infections [36].

There has been progress in the treatment of many inflammatory diseases, initially rheumatoid arthritis, and Crohn’s disease, then many others. TNF (Tumor Necrosis Factor) inhibitors have been an important step forward in the treatment of several chronic inflammatory diseases, especially rheumatoid arthritis, and Crohn’s disease. But these drugs are injectable and costly and have a potential for adverse effects, such as reactivation of latent tuberculosis. Furthermore, many patients treated with TNF inhibitors continue to experience chronic pain and so there is a big unmet need for cheaper, orally available drugs that reduce inflammation and pain [37].

The search for easily prepared, small molecular weight compounds, which can be delivered as an oral formulation continues. A hitherto mostly untapped wealth of novel compounds are natural products from plants, known to possess anti-inflammatory properties. Indeed, Cannabis sativa preparations and pure cannabinoids have been used to relieve symptoms of pain associated with several clinical disease conditions and ailments.

Previously we have reported the inflammatory disease modulating properties of CBD in several in vitro and in vivo systems and have demonstrated a potent ameliorating effect on the clinical signs of arthritis in a model of Rheumatoid Arthritis (RA) [38]. In a related study, our group has also established the potential of HU-320, a synthetic CBD derivative as a promising candidate for use in RA [39]. The anti-inflammatory activity of CBD and HU-320, as previously demonstrated by us in various experimental systems, has led us to explore the potential of CBG derivatives, new, chemically related cannabinoid compounds, which are much less studied, with the aim to evaluate therapeutic potential.

The synthesis of HUM-223, HUM-233 and HUM-234 uses CBG as starting material. CBG is formed in the plant (or by heating) from its precursor CBGA [40]. CBG acid is present in young plants in most Cannabis sativa varieties, including hemp, which is used for industrial purposes. Hemp is grown in many parts of the world, including Europe, for the production of textile fibers. Hence, CBG is potentially an inexpensive natural product. The synthetic pathway from CBG to the derivatives is a short, but relatively low yield one. In view of the simplicity of the reactions described herein, it is predicted that the yields can be increased.

The in-vivo assays used in the present study are well established and widely used in inflammation research [41,42]. The results indicate that HUM-223, HUM-233 and HUM-234 are anti-inflammatory candidates (and HUM-234 also anti-obesity), which we believe have the potential to be developed into therapeutic leads.

HUM-223 was able to significantly reduce swelling in all doses and exceed CBG’s effect at a dose of 10 mg/kg. In addition, it showed activity in reducing pain responses and reducing TNF-α levels. The latter effects were comparable to CBG.

We chose HUM-223 to be tested on the knee-arthritis model to complement the described paw arthritis model, as previously similar CBD derivatives, possessing DMH (dimethylheptyl) side chain were more active than pentyl chain derivatives in this model (unpublished results). We were able to prove that HUM-223 is indeed efficacious and doses of 5, 10 and 25 mg/kg have a comparable response to dexamethasone. A dose of 5 mg/kg HUM-223 was proved as the most effective dose out of the three doses tested. At this dose, the effect of HUM-223 was similar to dexamethasone, a known highly effective anti-inflammatory steroidal drug. Furthermore, HUM-223 was shown to reduce local gene expression of Elane, Mpo and Adamts4 at the endpoint of the experiment; enzymes upregulated in inflammatory arthritis, shown to play a central role in oxidant production, cartilage degradation and joint damage [43,44,45]. In line with our findings, CBG has previously been shown to decrease myeloperoxidase in the context of inflammatory bowel disorder [9], however to our knowledge there have been no previous reports of CBG/CBG analogs reducing neutrophil elastase or Adamts4.

Regarding HUM-233 and HUM-234, both showed anti-inflammatory activity comparable to CBG in all three assays. They were all able to reduce swelling, decrease evoked pain responses upon application of localized pressure on the inflamed paw. We observed an inverse dose-response trend upon increasing the dose of the compounds (Figure 5). The salt HUM-233 at a dose of 10 mg/kg does not equal the same dose of the free base. The inverse linear dose-response behavior of HUM-233 and HUM-234 could be therefore explained as a bell-shaped dose response curve of HUM-234 since the dosage of HUM-233 given represents a smaller dosage of the free amine. A bell-shaped dose-response for CBD has been documented, but for CBG or its derivatives, it has rarely been documented [15].

Of notable interest is the effect on weight gain in an established standard model of obesity. We chose HUM-234 to be assayed on diet-induced obesity, as we previously established a similar derivative of CBD, HU-435, could prevent weight gain in mice [46]. Indeed, HUM-234 prevented the high-fat diet-induced weight gain at a dose of 25 mg/kg. Obesity leads to metabolic abnormalities, so we examined the effect of HUM-234 on the serum levels of liver enzymes ALT, AST and liver damage. We found that serum levels of both ALT and AST were significantly lowered and observed significant amelioration of histological liver damage, with a healthy liver almost fully restored with the administration of 25 mg/kg HUM-234. Importantly, liver sparing was not observed with administration of CBG, suggesting that this novel derivative offers improved benefits over the parent compound for the treatment of obesity and metabolic disease.

The effect of HUM-234 on diet-induced obesity is of great interest to us. Obesity is an “epidemic” of the developed world [47]. It causes abnormal physiological metabolism, which leads to a series of physiological, psychological, and social problems. Additionally, obesity is an important risk factor for diseases such as hypertension [48], hyperlipemia [49], diabetes [13] and even cancer [50], and it is closely associated with the emergence of many chronic diseases [51]. The medications currently used for obesity are only approved for patients who are obese (BMI (body mass index) > 30), or overweight (BMI > 27) with one weight-related health issue, as they possess numerous side effects. The quest for an effective and safe treatment for obesity is ongoing.

We are aware of the limitations posed by the compounds’ biological behavior. All three compounds showed some variability in the concentrations most active in the swelling, pain sensation and TNF-α assays as did CBG. This is a known and well-documented trend in cannabis studies [52]. Moreover, the observed bell-shaped dose-response is unfavored when looking for new therapeutics. These challenges should be addressed in further development. However, the activity of HUM-234 in diet-induced obesity assay and in liver enzymes suggests that this may be a candidate therapeutic for obesity.

We believe that CBG derivatives HUM-223, HUM-233 and HUM-234 have the potential to be further developed as novel drug candidates for use in inflammatory conditions, and potentially also as anti-obesity treatment, where there is a huge unmet need.

## 4. Materials and Methods

### 4.1. NMR Spectroscopy

NMR data were collected on Varian Unity Inova 300 MHz spectrometer using the standard pulse sequences and processed with Agilent software.

### 4.2. Mass Spectrometry

The samples were analyzed by GC-MS in a Hewlett-Packard G1800 A GCD system with HP-5971 gas chromatograph with electron ionization detector. Ultra-low-bleed 5%-phenyl capillary column (28 mm × 0.25 mm (i.d.) × 0.25 μm film thickness) based on diphenyl methylsiloxane chemistry (HP-5MS; Agilent Technologies, Santa Clara, CA, USA) was used. Experimental conditions were: inlet, 250 °C; detector, 280 °C; splitless injection time; initial temperature, 90 °C; initial time, 3.00 min; rate, 25 °C/min; final temperature, 280 °C; helium flow rate, 1.0 mL/min. The software used was GCD Plus ChemStation.

LC-ESI-MS was done with Waters LC e2695 Separation Module equipped with reversed phase C18 column (Xselect^®^ CSH, 2.1 × 100 mm, 2.5 um, Waters (TC) Israel Ltd., Petah Tikva, Israel) connected to Waters 2489 UV/visible Detector and Waters QDa Detector. The Software used was MassLynx V4.2. Several gradients of Acetonitrile and water both containing 0.1% FA were developed for analysis starting from 0% to 100% Acetonitrile or 50% to 100% Acetonitrile. Eventually, the addition of Methanol to the gradient was proved effective for the analysis of our compounds and the final method of analysis was as follows: column temperature: 45 °C, UV detector: 225 nm, sampling rate 20 points/s; QDa detector: ES (+) *m*/*z* between 100 to 1000, cone voltage 2V, ES (−) *m*/*z* between 100 to 1000, cone voltage 2 V. Gradient: starting point: 15% acetonitrile (0.1% FA), 15% methanol (0.1% FA) and 70% water (5% acetonitrile and 0.1% FA) from 0 to 15 min. Then, 40% acetonitrile (0.1% FA), 40% methanol (0.1% FA) and 20% water (5% acetonitrile and 0.1% FA) from 15 to 22 min. Equilibration to starting conditions from 22 to 27 min. Flow rate: 0.25 mL/min. Probe temperature: 600 °C, source temperature: 120 °C, turbo temperature: 49 °C.

### 4.3. Chemical Synthesis

All the chemicals and solvents used were purchased from well-established commercial sources and used without any further purification procedures.

Newly synthesized cannabinoids and intermediate compounds were characterized by ^1^H NMR, ^13^C NMR and either GCMS or LC-ESI-MS.

#### 4.3.1. 1,1-Dimethylheptyl Cannabigerol (CBG-DMH)

1.84 gr (7.8 mmol) of DMHR are dissolved in 3.3 mL of dry DCM with 0.13 gr (0.78 mmol) of p-toluenesulfonic acid (PTSA). This solution is then cooled to 0 °C. Separately, 1.35 mL (7.8 mmol) of geraniol are dissolved in 2.6 mL of dry DCM and then cooled to 0 °C as well. The cold geraniol solution is then added dropwise with a high stir to the cold DMHR solution. The reaction is then stirred at RT for 45 min and quenched by the addition of sat. NaHCO_3_ solution. The water phase is then separated from the organic phase and the former is further extracted with DCM three times. The combined organic phase is washed with brine, dried over MgSO_4_ and evaporated. The crude product is then purified by silica gel column chromatography (TLC 20% EtOAc: Pet Ether). CBG-DMH is purified by repeated chromatography. CBG-DMH is obtained as a pale-yellow oil. Yield: 0.09 gr (30%). Analytical characteristics were in accordance with previously published literature [53].

*O*-Methyl-*O*-Acetoxy-CBG-DMH. (1) is prepared from CBG-DMH in two steps without purification in between. 1.67 gr (1.8 mmol) of CBG-DMH are dissolved in 10 mL of dry DMF under a nitrogen atmosphere. 0.37 gr (2.71 mmol) of potassium carbonate is added and the suspension is then allowed to stir at room temperature for 5 min. To this suspension, 60 µL (0.9 mmol) of methyl iodide is added. The reaction is allowed to stir overnight at room temperature. The reaction is then diluted with 10% *w*/*v* HCl to pH 1 and extracted three times with Et_2_O. The organic phase is washed with sat. NaHCO_3_ to pH 10 and with brine to neutral pH. It is then dried over MgSO_4_ and evaporated. Since chromatography of the crude did not efficiently separate the mono-methoxylated CBG-DMH from the di-methoxylated CBG-DMH, we decided to acetylate the free phenol that remained on the mono-methoxylated CBG-DMH. This method, in our hands, makes the chromatography much simpler and improves the overall yield. The 0.7 gr of crude methoxylation product are therefore carried to the next step without further purification.

The crude is dissolved in 10 mL of pyridine under nitrogen atmosphere with a catalytic amount of 4-dimethylaminopyridine (4-DMAP). 2 mL (18.2 mmol) of acetic anhydride are added slowly and the reaction is stirred at room temperature, monitoring by TLC. The reaction is worked up by diluting with EtOAc and washing the organic phase with 10% *w*/*v* HCl to pH 1. The aqueous phase is then extracted three times with EtOAc. The combined organic phase is then washed with sat. NaHCO_3_ to pH 8 and brine to neutral pH. The organic phase is dried over MgSO_4_ and evaporated. The crude product is purified by silica gel column chromatography (TLC 10% EtOAc: Pet Ether). Yield after two steps: 0.23 gr (30%). Compound **(1)** is obtained as yellow oil. 1H NMR (500 MHz, CDCl_3_) δ 6.75 (s, 1H), 6.63 (s, 1H), 5.18 (t, *J =* 6.9 Hz, 1H), 5.11 (t, *J =* 6.9 Hz, 1H), 3.85 (s, 3H), 3.27 (d, *J =* 7.0 Hz, 2H), 2.31 (s, 3H), 2.09 (t, 2H), 2.01 (t, 2H), 1.78 (s, 3H), 1.68 (s, 3H), 1.61 (s, 3H), 1.31 (s, 6H), 1.25 (q, *J =* 9.8, 7.9 Hz, 6H), 1.15 (d, *J =* 13.1 Hz, 2H), 0.89 (t, *J =* 6.9 Hz, 3H). 13C NMR (126 MHz, CDCl_3_) δ 169.36, 157.94, 149.38, 149.20, 134.76, 131.15, 124.40, 122.26, 119.52, 112.54, 106.22, 77.39, 77.14, 55.74, 44.59, 39.76, 37.86, 31.80, 30.06, 28.87, 26.72, 25.68, 24.64, 22.98, 22.73, 20.95, 17.67, 16.06, 14.12. GCMS: *m*/*z* 428 tR:13.67 min.

#### 4.3.2. 1″,1″-Dimethylheptyl-monomethoxycannabigerol (HUM-223)

0.52 gr (1.2 mmol) of (1) is dissolved in 15 mL of dry THF under a nitrogen atmosphere and cooled to 0 °C. Then, 0.49 gr (12.8 mmol) of LiAlH_4_ is added, and the reaction is heated to reflux and monitored by TLC. The reaction is cooled to room temperature and the LiAlH_4_ is neutralized first by dropwise addition of EtOAc followed by ice and 10% *w*/*v* HCl. The water phase is extracted three times with EtOAc. The combined organic phase is washed with sat. NaHCO_3_ and brine, dried over MgSO_4_ and evaporated. The crude product is purified by medium pressure liquid chromatography (TLC 10% EtOAc:Pet Ether). HUM-223 is obtained as a colorless oil. Yield: 0.25 gr (54%). 1H NMR (300 MHz, CDCl_3_) δ 6.48 (1H, s), 6.46 (1H, s), 5.31–5.29 (1H, m), 5.08–5.06 (1H, m), 3.83 (3H, s), 3.43 (2H, d, *J =* 6.9 Hz), 2.13–2.07 (2H, m), 1.83 (3H, s), 1.75–1.66 (1H, m), 1.69 (3H, s), 1.61 (3H, s), 1.59–1.54 (1H, m), 1.3 (12H, s), 1.22–1.09 (2H, m), 0.87 (3H, t, *J =* 6.9 Hz). 13C NMR (75 MHz, CDCl_3_) δ 157.45, 155.13, 149.65, 137.93, 131.85, 123.95, 122.24, 112.01, 106.89, 101.15, 55.78, 44.56, 39.75, 37.75, 31.83, 30.09, 28.98, 26.48, 25.73, 24.69, 22.74, 22.11, 17.72, 16.16. 14.15. GCMS: *m*/*z* 386 t_R_: 13.7 min.

#### 4.3.3. Monomethoxycannabigeroyl-3-morpholinopropraonate Maleate (HUM-233)

0.343 gr (0.72 mmol) of HUM-234 are dissolved in 40 mL of isopropyl alcohol (IPA). Then, 83.5 mg (0.72 mmol) of maleic acid are added and the reaction is stirred at room temperature for 2.5 h. IPA is evaporated and HUM-233 is recrystallized from EtOAc and ether. HUM-233 is obtained as crystalline white solid. Yield: 0.32 gr (75%). 1H NMR (300 MHz, CDCl_3_) δ 6.59 (s, 1H), 6.46 (s, 1H), 6.33 (s, 2H), 5.09–4.96 (m, 2H), 4.04–3.89 (m, 4H), 3.82 (s, 3H), 3.40 (t, *J =* 7.0 Hz, 2H), 3.26–3.05 (m, 4H), 2.56 (t, *J =* 7.8 Hz, 2H), 2.10–1.96 (m, 2H), 1.96–1.85 (m, 2H), 1.70 (s, 3H), 1.64 (s, 3H), 1.56 (s, 3H), 1.40–1.24 (m, 4H), 0.89 (t, *J =* 6.8 Hz, 3H). LCMS:ES (+) *m*/*z* 472 [M + H], 494 [M + Na] tR: 7.26 min, ES (−) *m*/*z* 115 [M − H], 231 [2M − H] tR: 1.91 min.

#### 4.3.4. Monomethoxycannabigeroyl-3-morpholinoproprionate (HUM-234)

0.432 mL of methyl morpholinoproprionate are dissolved in 4 mL of 1,4-dioxane, 2.16 mL of water and 1.08 mL of 3N aqueous sodium hydroxide. The reaction is stirred at room temperature for 4.5 h and then the solvent is evaporated. The solids are suspended in 40 mL of dry DCM under nitrogen atmosphere and small amount of MgSO_4_ is added to insure the absence of water in the reaction flask. Then, 0.889 gr of O-monomethyl cannabigerol (2) are added, followed by 0.04 gr of pyrrolidinopyridine. The reaction is then stirred at room temperature for 5 min. 0.56 gr of *N*,*N*-dicyclohexylcarbodiimide (DCC) are added and the reaction is stirred at room temperature overnight. The solids are filtered, and the solution is evaporated. The crude product is purified by silica gel column chromatography (TLC 20% EtOAc:Pet Ether). HUM-234 is obtained as yellow oil. Yield: 0.25 gr (20%). ^1^H NMR (300 MHz, CDCl_3_) δ 6.57 (s, 1H), 6.50 (s, 1H), 5.19–4.99 (m, 2H), 3.80 (s, 3H), 3.72 (t, *J =* 4.6 Hz, 4H), 3.25 (d, *J =* 6.9 Hz, 2H), 2.88–2.66 (m, 4H), 2.55–2.48 (m, 5H), 2.11–2.00 (m, 2H), 1.99–1.89 (m, 2H), 1.73 (s, 3H), 1.65 (s, 3H), 1.57 (s, 3H), 1.39–1.24 (m, 4H), 0.91 (d, *J =* 6.0 Hz, 3H). ^13^C NMR (75 MHz, CDCl_3_) δ 170.89, 158.12, 149.31, 145.98, 141.99, 134.79, 131.18, 124.32, 122.26, 119.72, 114.34, 108.53, 66.91, 55.70, 54.03, 53.42, 39.75, 35.96, 32.30, 31.57, 30.92, 26.69, 25.71, 22.85, 22.57, 17.69, 16.11, d14.08. LCMS: ES (+) *m*/*z* 472 [M + H], 494 [M + Na] t_R_: 7.26 min.

### 4.4. Biological Evaluation

#### 4.4.1. Animals

Female Sabra mice (for the swelling, pain and TNF-α experiments) and C57Bl6 (for obesity experiments, a line that was shown to gain much weight under HFD conditions), 7–8 weeks old, were maintained in the specific-pathogen-free unit of the Hadassah Medical School, Hebrew University, Jerusalem, Israel. The experimental protocols were approved by the Institutional Animal Care Ethics Committee (permission # MD-20-16042-5). The animals were maintained at a constant temperature (20–21 °C) and a 12-h light/12-h dark cycle and were provided a standard pellet diet with water *ad libitum*. The mice were acclimatized in the animal facility for at least 2 weeks before the experiments. The data presented in Figures are representatives of 2 separate experiments.

#### 4.4.2. Induction and Treatment of Paw Inflammation (Paw ZIA)

Inflammation was induced by injection of 40 uL of a suspension of 1.5% *w*/*v* zymosan A (Sigma-Aldrich Israel Ltd., Rehovot, Israel) in saline into the subplantar surface of the right hind paw of the mice. This was followed immediately by an intraperitoneal injection of the test compound. For injection, the compounds were dissolved in a vehicle containing ethanol:Cremophore:saline at a ratio of 1:1:18. Paw swelling and pain perception were assessed after 2, 6 and 24 h. Blood was collected after 24 h for analysis of TNFα serum levels.

#### 4.4.3. Evaluation of Edema

Calibrated calipers were used to measure paw swelling (thickness) 2, 6 and 24 h after injection of zymosan.

#### 4.4.4. Pain Assay

Pain at 2, 6 and 24 h after zymosan injection was assessed by the von Frey nociceptive filament assay, where 1.4–60 g filaments, corresponding to 4.17–5.88 log of force, was used to test the sensitivity of the swollen paw. The untreated hind paw served as a control. The measurements were performed in a quiet room and the animals were handled for 10 s before the test. A trained investigator then applied the filament, poking the middle of the hind paw to provoke a flexion reflex, followed by a clear finch response after paw withdrawal. Filaments of increasing size were each applied for about 3–4 s. The mechanical threshold force in grams was defined as the lowest force required to obtain a paw retraction response.

#### 4.4.5. Measurement of TNFα

Blood was collected 24 h after zymosan injection, and the sera were assayed for TNFα using a mouse TNFα ELISA kit (R&D Systems, Minneapolis, MN, USA), according to the manufacturer’s instructions.

#### 4.4.6. Zymosan Induced Arthritis of the Knee

All experimental procedures were approved by the Ethical Review Process Committee and the UK Home Office, in accordance with the 1986 Animals (Scientific Procedures) Act (permission # 30/3441). Male C57BL/6J mice aged 8–10 weeks were used. Mice were housed in ventilated cages, maintained at 21 °C ± 2 °C and a 12-h light/12-h dark cycle, with food and water available ad libitum. Mice were anesthetized with 2% isoflurane and both knees were shaved. ZIA was induced by intra-articular injection of 180 µg of Zymosan A (Sigma) suspended in PBS as previously described [9]. Left knee joints received a vehicle control injection. For IVIS imaging, mice received an intravenous injection of 4 nmol Neutrophil Elastase 680 FAST imaging probe (Perkin Elmer, Waltham, MA, USA) and were imaged 4 h post intravenous injection using the IVIS Spectrum (Perkin Elmer) [54]. Images were analyzed using Living Image 4.7 software (Perkin Elmer) to obtain the average fluorescence intensities of a circular region of interest encompassing the knee joint. Mice were humanely culled 8 h post zymosan administration and knee joints were snap-frozen for gene expression analysis. RNA was extracted using the TRIzol method, as previously described [54]. Reverse transcription of 1 ug of total RNA was conducted using a High-Capacity cDNA Reverse Transcription Kit (Thermo Fisher Scientific) with random primers and following the manufacturer’s protocol. Real-time PCR was carried out using the power SYBR Green Master Mix in a real-time PCR system (Thermo Fisher Scientific, Waltham, MA, USA). Data are expressed as relative units calculated by 2−ΔΔCt by normalization relative to RPL32 and to fold change over vehicle-treated control samples.

#### 4.4.7. Diet-Induced Obesity

Mice were fed with a standard diet (STD) or high-fat diet (HFD) for 7 days before the beginning of the injections. On day 7 they were divided into the groups: STD, HFD, HFD+CBG and HFD+HUM-234, and treated for 35 days. The mice were weighed every week.

#### 4.4.8. Liver Injury

At the end of the diet-induced obesity experiment, the mice were sacrificed. The livers were removed, fixed with buffer formalin and stained with haematoxylin and eosin, for microscope evaluation. Paraffin sections of 4–5 µm thickness, were stained with hematoxylin and eosin. For microscopic evaluation, sections were examined (×40).

#### 4.4.9. Determination of ALT and AST Levels

The levels of two aminotransferases, alanine aminotransferase (ALT) and aspartate aminotransferase (AST), were assayed in the sera of mice at the end of diet-induced obesity experiment with or without HUM-234 treatment, by ALT and AST strips respectively (Refloram-Mannheim GmbH, Mannheim, Germany) and quantitated by an automated analyzer (Reflotran Plus, Roche, Basel, Switzerland).

### 4.5. Statistical Analyses

Statistical analysis was performed with GraphPad Prism software. Statistical analysis details are listed under each figure. The results are presented as value ± SE (standard error). In rare cases where all the measurements give the same values, no SE bar is presented, as no error can be measured. * *p*-value < 0.05, ** *p*-value < 0.01 and *** *p*-value < 0.001 when comparing to a control group. # *p*-value < 0.05 when comparing between the different dosages of the tested compounds, or when comparing them to CBG, the exact comparisons are listed on Figures’ legends.

## Data Availability

The data presented in this study are available on request from the corresponding author.

## Sample Availability

Samples of the compounds are not available from the authors.
